# Half-marathoners are younger and slower than marathoners

**DOI:** 10.1186/s40064-016-1704-9

**Published:** 2016-01-26

**Authors:** Beat Knechtle, Pantelis T. Nikolaidis, Matthias A. Zingg, Thomas Rosemann, Christoph A. Rüst

**Affiliations:** Gesundheitszentrum St. Gallen, St. Gallen, Switzerland; Institute of Primary Care, University of Zurich, Zurich, Switzerland; Department of Physical and Cultural Education, Hellenic Army Academy, Athens, Greece

**Keywords:** Women, Men, Master runner, Age group

## Abstract

Age and performance trends of elite and recreational marathoners are well investigated, but not for half-marathoners. We analysed age and performance trends in 508,108 age group runners (125,894 female and 328,430 male half-marathoners and 10,205 female and 43,489 male marathoners) competing between 1999 and 2014 in all flat half-marathons and marathons held in Switzerland using single linear regression analyses, mixed-effects regression analyses and analyses of variance. The number of women and men increased across years in both half-marathons and marathons. There were 12.3 times more female half-marathoners than female marathoners and 7.5 times more male half-marathoners than male marathoners. For both half-marathons and marathons, most of the female and male finishers were recorded in age group 40–44 years. In half-marathons, women (10.29 ± 3.03 km/h) were running 0.07 ± 0.06 km/h faster (p < 0.001) than men (10.22 ± 3.06 km/h). Also in marathon, women (14.77 ± 4.13 km/h) were running 0.28 ± 0.16 km/h faster (p < 0.001) than men (14.48 ± 4.07 km/h). In marathon, women (42.18 ± 10.63 years) were at the same age than men (42.06 ± 10.45 years) (p > 0.05). Also in half-marathon, women (41.40 ± 10.63 years) were at the same age than men (41.31 ± 10.30 years) (p > 0.05). However, women and men marathon runners were older than their counterpart half-marathon runners (p < 0.001). In summary, (1) more athletes competed in half-marathons than in marathons, (2) women were running faster than men, (3) half-marathoners were running slower than marathoners, and (4) half-marathoners were younger than marathoners.

## Background

Marathon running is a very popular sport event held all over the world with an increasing number of races and successful finishers over the last years (Ahmadyar et al. 2015; Jokl et al. 2004; Knechtle et al. 2015b; Lehto 2015; Leyk et al. 2007; Lepers and Cattagni 2012). For example, in the USA, there were more than ~1200 marathons held in 2014 compared to ~300 marathons in 2000 (www.runningusa.org/2015-national-runner-survey). The number of successful marathon finishers increased from ~25,000 in 1976 to the all-time high of ~550,600 in 2014. However, in the USA, more runners competed in half-marathons than in marathons. The number of half-marathoners increased from ~303,000 in 1990 to the all-time high of ~2,046,600 in 2014. That was, the number of half-marathoners was in 2014 ~3.7 times higher than the number of marathoners in the USA.

Considering the popularity of half-marathon races, several studies have examined recently many issues (i.e. mostly health-related) in this sport event by comparing it with corresponding trends in full marathon running (De Gonzalo-Calvo et al. 2015; Hart 2013; Jassal et al. 2009; Kim et al. 2012; Reihmane et al. 2013). In a study on the effect of aerobic exercise on systemic inflammation, half-marathoners showed lower levels of inflammatory parameters after the race compared to marathoners (De Gonzalo-Calvo et al. 2015). In addition, it has been shown that the increase in interleukin-6, tumour necrosis factor-alpha and matrix metalloproteinase-9 after the race was smaller in half-marathoners than in marathoners (Reihmane et al. 2013). In a study on the effect of aerobic exercise on cardiac injury markers, half-marathoners demonstrated lower elevations in creatinine kinase, myoglobin and cardiac troponin T compared to marathoners (Jassal et al. 2009). Moreover, the overall incidence of cardiac arrests in the USA from 2000 to 2010 was lower in half-marathoners than in marathoners (Hart 2013; Kim et al. 2012). These studies focusing on health-related aspects have highlighted certain differences in the response of various physiological mechanisms to aerobic exercise between half-marathon and marathon running.

The abovementioned studies on differences between half-marathoners and marathoners have enhanced our understanding of the responses of certain physiological mechanisms to aerobic exercise of various durations. However, only a few data are available about major aspects (e.g. age, sex and race speed) related to performance differences between these two popular running events (Leyk et al. 2007; Zillmann et al. 2013). These studies investigated only a limited time frame or a limited sample of athletes. Leyk et al. (2007) analysed race times and ages of half-marathoners and marathoners for 3 years (2002–2005) and Zillmann et al. (2013) performed a field study on male half-marathoners and marathoners. The knowledge of half-marathon runners’ basic characteristics such as age, sex, participation and performance trends might help coaches, fitness trainers and sports scientists to improve their understanding of half-marathon’s demands compared to the corresponding profile of a full marathon. Therefore, the aim of this study was to compare age, sex, participation and performance between female and male half-marathoners and marathoners in a sample of more than 500,000 runners competing in half-marathons and marathons held in one country during a period of 15 years.

## Materials and methods

### Ethics approval

The study was approved by the Institutional Review Board of St. Gallen, Switzerland, with waiver of the requirement for informed consent given that the study involved the analysis of publicly available data.

### Data sampling and data analysis

All marathons and half-marathons held in Switzerland were identified by using data from ‘Laufkalender Schweiz’ (www.laufkalender.ch). In Switzerland, all running races started in 1999 to record race times with an electronic chip system and all race results became available in this year on the websites of the specific races. Of all recorded races, only those half-marathons and marathons were considered which were held on a road, not on a trail. Only flat marathons were considered and no mountain marathons were included. For all considered races, start and finish had to be on the same altitude. Athletes with missing age were excluded from data analysis. In order to avoid a selection bias due to a limitation to top runners (e.g. annual fastest, annual ten fastest), we considered all finishers. Race times recorded in the ranking lists were converted to running speed (km/h) using race distance (km) and race time (h:min).

### Statistical analysis

Each set of data was tested for normal distribution (D’Agostino and Pearson omnibus normality test) and for homogeneity of variances (Levene’s test) before statistical analyses. Differences in the participation of long-distance runners by sex to half-marathon and marathon running were examined using Chi square (χ^2^) test. Trends in participation across calendar years were analyzed using regression with linear growth equation models. A mixed-effects regression model with finisher as random variable to consider finishers who completed several races was used to analyze changes in performance of finishers across years. We included sex, centered age, squared centered age and calendar year as fixed variables. Sex difference was calculated as sex difference = (running speed in women − running speed in men)/running speed in men × 100, where running speed in men was defined as 100 %. Multiple groups were compared using one-way analysis of variance (ANOVA) with subsequent Tukey’s post hoc multiple comparison test, with a single pooled variance. Statistical analyses were performed using IBM SPSS Statistics (Version 22, IBM SPSS, Chicago, IL, USA) and GraphPad Prism (Version 6.01, GraphPad Software, La Jolla, CA, USA). Significance was accepted at p < 0.05 (two-sided for *t*-tests). Data in the text are given as mean ± standard deviation (SD). Data in the figures are given as mean ± 95 % confidence interval (CI) for box–whisker-plots and mean ± SD for trends across time.

## Results

Data from a total of 508,108 (i.e. 125,894 female and 328,430 male half-marathoners and 10,205 female and 43,489 male marathoners) athletes could be considered. There were 12.3 times more female half-marathoners than female marathoners and 7.5 times more male half-marathoners than male marathoners. There was a statistically significant association between the sex of long-distance runners and the format of the race [χ^2^(1) > 40.35 × 10^6^, p < 0.001]. That was, compared to men, women participated more in half-marathon than in marathon running.

### Participation

In half-marathons, the number of women (r^2^ = 0.98, p < 0.0001) and men (r^2^ = 0.98, p < 0.0001) increased significantly. Similarly, the number of women (r^2^ = 0.46, p = 0.0041) and men increased significantly (r^2^ = 0.51, p = 0.0019) in marathons (Table 1). The men-to-women ratio decreased significantly in half-marathons (r^2^ = 0.71, p < 0.0001) but remained unchanged in marathons (r^2^ = 0.21, p = 0.075) (Table 2). For both half-marathons and marathons, most female and male finishers were recorded in age group 40–44 years (Table 3).

**Table 1:** The number of female and male finishers in half-marathon and marathon

Year	Half-marathon		Marathon	
	Women	Men	Women	Men
1999	1674	6093	156	512
2000	2704	9793	174	807
2001	3283	10,104	282	923
2002	4321	12,882	255	905
2003	5057	15,178	542	2485
2004	6537	17,275	658	2866
2005	6704	17,249	844	3648
2006	7233	18,583	781	3139
2007	6686	20,879	1073	4346
2008	9180	23,254	992	3918
2009	10,625	25,450	879	3908
2010	10,332	27,675	680	3453
2011	11,782	30,035	785	3453
2012	11,615	27,700	693	3126
2013	13,825	32,555	692	3199
2014	14,336	33,725	719	2801
Total	125,894	328,430	10,205	43,489

**Table 2:** The men-to-women ratio for half-marathon and marathon

Year	Half-marathon	Marathon
1999	3.63	3.28
2000	3.62	4.63
2001	3.07	3.27
2002	2.98	3.54
2003	3.00	4.58
2004	2.64	4.35
2005	2.57	4.32
2006	2.56	4.01
2007	3.12	4.05
2008	2.53	3.94
2009	2.39	4.44
2010	2.67	5.07
2011	2.54	4.39
2012	2.38	4.51
2013	2.35	4.62
2014	2.35	3.89

**Table 3:** Distribution of the athletes regarding the age groups

Age group	Half-marathon		Marathon	
	Women	Men	Women	Men
18–24	5709	15,231	405	1632
25–29	11,111	29,003	859	3507
30–34	16,343	43,109	1255	5516
35–39	20,796	55,542	1598	7100
40–44	24,286	62,976	1967	8461
45–49	20,839	53,616	1653	7232
50–54	13,619	34,992	1204	4918
55–59	7458	19,072	664	2685
60–64	3652	9419	344	1434
65–69	1460	3865	173	639
70–74	482	1216	67	271
75–79	114	324	14	74
80–84	19	52	2	13
85–89	4	11		6
90–94	2	2		1

### Performance

**Fig. 1:**
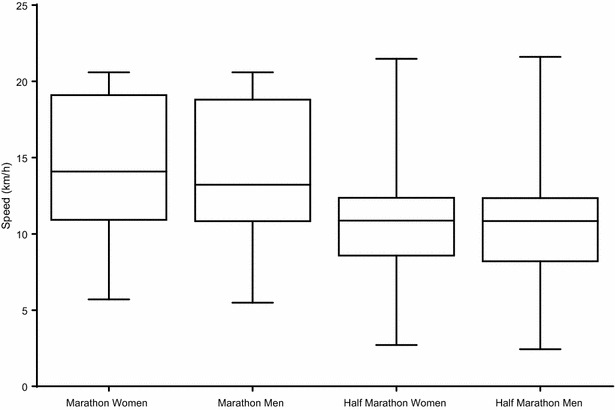
Box–whisker-plot for running speeds in female and male half-marathoners and marathoners. Data are presented as mean ± 95 % confidence interval

Figure 1 shows the box–whisker-plots for running speed for female and male half-marathoners and marathoners. In half-marathons, women were running at 10.29 ± 3.03 km/h and men at 10.22 ± 3.06 km/h. Women were running 0.07 ± 0.06 km/h faster than men (p < 0.001). Female marathoners were running at 14.77 ± 4.13 km/h and male marathoners at 14.48 ± 4.07 km/h. Women were running 0.28 ± 0.16 faster than men (p < 0.001). When marathoners and half-marathoners were compared, female marathoners were running 4.47 ± 1.12 km/h faster than female half-marathoners (p < 0.001) and male marathoners were running 4.26 ± 0.99 km/h faster than male half-marathoners (p < 0.001).

**Fig. 2:**
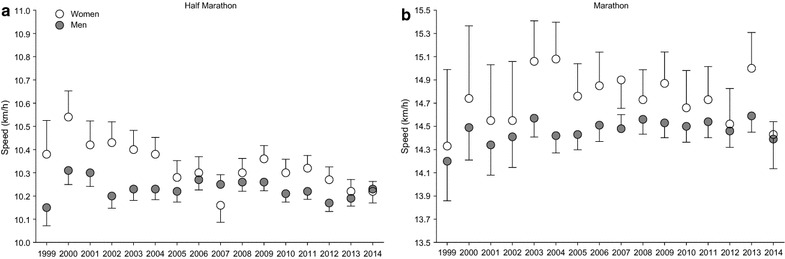
Running speed across years in half-marathoners and marathoners. Data are presented as mean ± SD

Running speed decreased significantly across years in female half-marathoners (r^2^ = 0.55, p = 0.0010), but remained unchanged in male half-marathoners (r^2^ = 0.05, p = 0.38) (Fig. 2a). In female (r^2^ = 0.00, p = 0.80) and male (r^2^ = 0.24, p = 0.051) marathoners, running speed remained unchanged (Fig. 2b).

Regarding running speed for female (Table 4) and male (Table 5) age group half-marathoners, running speed decreased significantly in age groups 25–29 to 55–59 years (Table 6). Women were faster than men in age groups 25–29 to 35–39, 45–49 and 50–54 years. In marathon races (Tables 7, 8), running speed increased significantly in age group 80–84 years (Table 9). Women were faster than men in age groups 40–44, 50–54 and 55–59 years.

**Table 4:** Running speed (km/h, mean ± SD) for female age group half-marathoners

Year	18–24	25–29	30–34	35–39	40–44	45–49	50–54	55–59	60–64	65–69	70–74	75–79	80–84	85–89	90–94
1999	11.03 ± 3.33	10.80 ± 3.09	10.51 ± 3.06	10.57 ± 3.05	10.36 ± 3.11	10.26 ± 3.00	10.44 ± 2.74	9.73 ± 3.09	9.64 ± 2.76	9.23 ± 2.30	7.51 ± 3.39	10.12 ± 0.13		5.17	
2000	11.03 ± 2.86	10.85 ± 2.93	10.68 ± 2.89	10.77 ± 3.07	10.43 ± 3.09	10.36 ± 3.05	10.27 ± 2.95	10.45 ± 2.96	10.61 ± 2.25	10.08 ± 2.48	9.28 ± 1.71	7.39 ± 0.14			
2001	10.75 ± 2.98	11.06 ± 3.04	10.61 ± 3.07	10.53 ± 3.04	10.58 ± 3.07	10.18 ± 2.91	10.03 ± 3.05	9.94 ± 2.87	10.02 ± 2.75	9.08 ± 2.54	9.19 ± 2.03	9.52 ± 1.75	9.13		
2002	10.84 ± 2.94	10.88 ± 3.03	10.80 ± 3.02	10.50 ± 3.05	10.27 ± 3.15	10.21 ± 3.05	10.30 ± 2.91	10.13 ± 2.77	9.99 ± 2.56	9.86 ± 2.69	10.02 ± 1.49	9.35 ± 0.78	10.92 ± 0.27		
2003	11.08 ± 3.13	10.59 ± 3.10	10.57 ± 3.12	10.55 ± 3.07	10.07 ± 3.06	10.44 ± 3.00	10.35 ± 2.78	10.15 ± 2.82	10.11 ± 2.50	10.21 ± 2.25	9.79 ± 2.01	9.46 ± 1.59			
2004	10.98 ± 3.18	10.84 ± 3.12	10.47 ± 3.05	10.53 ± 3.03	10.33 ± 3.05	10.19 ± 2.96	10.29 ± 2.94	10.05 ± 2.74	9.79 ± 2.85	9.48 ± 2.69	10.67 ± 1.45	9.40 ± 0.80	9.70	7.08	
2005	10.60 ± 3.11	10.55 ± 3.16	10.48 ± 3.16	10.40 ± 3.09	10.28 ± 3.09	10.16 ± 2.99	10.10 ± 2.91	9.89 ± 2.75	9.68 ± 2.87	9.91 ± 2.66	9.40 ± 2.00	10.37 ± 1.44	8.36 ± 2.06		
2006	10.82 ± 3.04	10.50 ± 3.08	10.49 ± 3.08	10.59 ± 2.93	10.18 ± 3.14	10.12 ± 2.99	10.18 ± 2.89	10.05 ± 2.81	9.76 ± 2.80	9.38 ± 2.69	9.80 ± 1.96	7.15 ± 3.03			6.41 ± 4.68
2007	10.67 ± 3.02	10.59 ± 3.10	10.31 ± 3.10	10.45 ± 3.13	9.93 ± 3.09	10.06 ± 3.03	9.87 ± 3.09	10.05 ± 2.73	9.62 ± 2.84	9.36 ± 2.73	8.31 ± 2.74	8.27 ± 1.86	7.41	9.30	
2008	10.87 ± 3.06	10.50 ± 3.16	10.58 ± 3.05	10.52 ± 3.05	10.14 ± 3.04	10.28 ± 2.99	10.01 ± 2.94	9.85 ± 2.86	9.74 ± 2.86	9.81 ± 2.64	9.75 ± 2.13	8.74 ± 2.20	8.22 ± 0.31		
2009	10.86 ± 2.92	10.60 ± 3.05	10.57 ± 3.13	10.38 ± 3.02	10.30 ± 3.06	10.31 ± 2.99	10.14 ± 3.01	10.15 ± 2.85	9.88 ± 2.69	10.01 ± 2.55	9.69 ± 1.93	10.96 ± 1.67	8.62 ± 1.09		
2010	10.81 ± 3.01	10.60 ± 3.10	10.63 ± 3.09	10.41 ± 3.05	10.23 ± 3.07	10.01 ± 3.01	10.21 ± 2.99	9.95 ± 2.80	9.89 ± 2.70	9.92 ± 2.40	9.46 ± 2.42	8.77 ± 2.20	7.51		
2011	10.62 ± 3.17	10.65 ± 3.09	10.56 ± 3.05	10.48 ± 3.05	10.27 ± 3.11	10.27 ± 3.01	9.92 ± 2.98	10.03 ± 2.88	9.87 ± 2.87	10.07 ± 2.34	8.88 ± 2.49	8.86 ± 2.57	9.49		
2012	11.09 ± 3.06	10.55 ± 3.04	10.56 ± 3.14	10.32 ± 3.03	10.20 ± 3.05	10.08 ± 3.05	9.99 ± 3.01	10.07 ± 2.76	9.79 ± 2.79	9.78 ± 2.45	9.40 ± 2.16	8.98 ± 1.79	7.09 ± 5.03	7.69	
2013	11.03 ± 3.02	10.63 ± 3.07	10.33 ± 3.16	10.26 ± 3.06	10.14 ± 3.12	10.11 ± 3.04	10.01 ± 2.96	9.89 ± 2.87	9.84 ± 2.89	9.57 ± 2.69	9.66 ± 2.26	8.69 ± 2.36	10.35 ± 0.23		
2014	10.87 ± 2.95	10.56 ± 3.09	10.40 ± 3.01	10.33 ± 3.14	10.21 ± 3.13	10.13 ± 2.93	9.88 ± 3.05	9.86 ± 2.93	9.70 ± 2.77	9.80 ± 2.38	9.40 ± 2.38	8.63 ± 1.66

**Table 5:** Running speed (km/h, mean ± SD) for male age group half-marathoners

Year	18–24	25–29	30–34	35–39	40–44	45–49	50–54	55–59	60–64	65–69	70–74	75–79	80–84	85–89	90–94
1999	10.87 ± 3.11	10.89 ± 2.88	10.33 ± 3.16	10.26 ± 3.14	10.07 ± 3.17	10.03 ± 3.10	9.82 ± 3.14	9.45 ± 3.08	9.77 ± 2.90	9.61 ± 2.83	8.45 ± 2.81	10.36 ± 2.05	9.51 ± 1.67	7.15	
2000	10.66 ± 3.14	10.77 ± 3.10	10.56 ± 3.08	10.38 ± 3.15	10.10 ± 3.12	10.32 ± 2.98	10.10 ± 2.98	10.15 ± 2.79	9.70 ± 2.85	9.66 ± 2.59	8.40 ± 2.76	7.96 ± 2.73			
2001	10.88 ± 3.01	10.73 ± 3.01	10.54 ± 3.08	10.28 ± 3.26	10.28 ± 3.05	10.26 ± 2.96	9.99 ± 2.97	9.85 ± 2.80	9.82 ± 2.92	9.16 ± 2.82	8.34 ± 2.39	8.44 ± 0.76		7.02	6.81
2002	10.87 ± 3.07	10.58 ± 3.07	10.38 ± 3.14	10.09 ± 3.10	10.13 ± 3.11	10.16 ± 3.03	9.99 ± 2.94	10.06 ± 2.84	9.95 ± 2.83	9.54 ± 2.77	9.42 ± 2.28	9.21 ± 1.80	8.68 ± 0.06		
2003	10.94 ± 2.92	10.43 ± 3.06	10.33 ± 3.17	10.38 ± 3.14	10.23 ± 3.07	10.08 ± 3.10	10.10 ± 2.96	9.99 ± 2.89	9.60 ± 2.87	9.57 ± 2.79	9.11 ± 2.60	8.32 ± 2.81	9.95 ± 0.82	8.70	
2004	10.80 ± 3.15	10.49 ± 3.08	10.33 ± 3.17	10.26 ± 3.12	10.24 ± 3.10	10.16 ± 2.99	10.02 ± 2.93	9.91 ± 2.81	9.93 ± 2.86	9.76 ± 2.49	9.27 ± 2.64	9.64 ± 1.76	9.24 ± 1.58	7.63 ± 1.77	
2005	11.01 ± 3.07	10.49 ± 3.15	10.33 ± 3.13	10.23 ± 3.15	10.17 ± 3.13	10.04 ± 3.06	10.16 ± 2.90	10.08 ± 2.84	9.67 ± 2.85	9.79 ± 2.48	9.62 ± 2.14	8.51 ± 2.43	8.23	9.94 ± 2.36	
2006	10.93 ± 3.14	10.74 ± 3.04	10.48 ± 3.12	10.33 ± 3.10	10.19 ± 3.14	10.02 ± 3.09	10.11 ± 2.95	10.04 ± 2.88	9.95 ± 2.75	9.69 ± 2.63	8.77 ± 2.37	9.56 ± 2.12	9.83 ± 1.59		
2007	10.75 ± 3.01	10.62 ± 3.12	10.38 ± 3.10	10.34 ± 3.17	10.26 ± 3.05	10.14 ± 3.10	9.93 ± 3.00	10.03 ± 2.82	9.75 ± 2.82	9.52 ± 2.39	9.42 ± 2.40	8.33 ± 2.53	8.05	8.04	7.21
2008	10.90 ± 2.96	10.59 ± 3.04	10.40 ± 3.09	10.35 ± 3.10	10.20 ± 3.11	10.14 ± 3.05	10.09 ± 3.02	9.92 ± 2.80	9.84 ± 2.82	9.55 ± 2.69	9.45 ± 2.21	8.76 ± 2.73	8.51		
2009	10.91 ± 3.12	10.61 ± 3.02	10.37 ± 3.15	10.38 ± 3.07	10.23 ± 3.08	10.16 ± 3.03	10.03 ± 3.01	9.94 ± 2.80	9.79 ± 2.78	9.69 ± 2.59	9.34 ± 2.32	8.50 ± 1.73	8.11 ± 1.69		
2010	10.75 ± 3.10	10.53 ± 3.12	10.38 ± 3.11	10.41 ± 3.09	10.11 ± 3.10	10.07 ± 3.06	10.04 ± 3.00	9.88 ± 2.89	9.77 ± 2.85	9.52 ± 2.64	9.15 ± 2.27	9.79 ± 1.85	8.29 ± 2.05		
2011	10.70 ± 3.05	10.56 ± 3.10	10.40 ± 3.12	10.31 ± 3.13	10.11 ± 3.08	10.13 ± 3.06	10.07 ± 2.99	9.90 ± 2.88	9.86 ± 2.83	9.52 ± 2.58	9.47 ± 2.31	9.56 ± 2.09	8.68 ± 2.07		
2012	10.83 ± 3.05	10.67 ± 3.05	10.32 ± 3.18	10.28 ± 3.13	10.12 ± 3.12	10.02 ± 3.06	9.94 ± 2.96	9.79 ± 2.91	9.74 ± 2.75	9.43 ± 2.70	9.33 ± 2.67	8.79 ± 2.51	8.02 ± 3.08	9.90	
2013	10.87 ± 3.15	10.47 ± 3.13	10.37 ± 3.17	10.31 ± 3.14	10.09 ± 3.17	10.12 ± 3.03	9.96 ± 3.04	9.85 ± 2.90	9.66 ± 2.87	9.63 ± 2.68	9.28 ± 2.48	8.97 ± 2.35	8.72 ± 1.44		
2014	10.81 ± 3.03	10.56 ± 3.11	10.38 ± 3.14	10.31 ± 3.13	10.21 ± 3.16	10.15 ± 3.05	9.94 ± 2.96	9.88 ± 2.94	9.70 ± 2.87	9.70 ± 2.62	9.39 ± 2.49	9.49 ± 1.40	8.15 ± 3.25	7.66

**Table 6:** Results of the mixed-effects regression analyses for running speed in half-marathon

Parameter	Estimate	SE	df	t	*p*
18–24 years					
Constant term	6.296428	9.049231	16,363.659	0.696	0.487
[Sex = female]	0.049958	0.052227	16,454.169	0.957	0.339
Calendar year	−0.001075	0.004398	16,470.927	−0.244	0.807
Cage	−0.672955	0.196899	12,995.658	−3.418	0.001
Cage^2^	−0.016791	0.004985	13,014.387	−3.369	0.001
25–29 years					
Constant term	26.794555	6.117458	28,544.800	4.380	<0.0001
[Sex = female]	0.122531	0.039147	29,404.862	3.130	0.002
Calendar year	−0.007846	0.002974	28,838.319	−2.638	0.008
Cage	0.092807	0.187007	22,771.577	0.496	0.620
Cage^2^	0.004071	0.006706	22,735.366	0.607	0.544
30–34 years					
Constant term	12.205098	4.717605	40,495.293	2.587	0.010
[Sex = female]	0.130067	0.033106	41,901.765	3.929	<0.0001
Calendar year	−0.001042	0.002342	40,566.839	−0.445	0.656
Cage	−0.081075	0.092362	32,386.180	−0.878	0.380
Cage^2^	−0.005457	0.005138	32,359.413	−1.062	0.288
35–39 years					
Constant term	10.270803	.028546	65,078.789	359.801	<0.0001
[Sex = female]	.243288	.057024	66,997.713	4.266	<0.0001
Calendar year	−.015718	.006081	41,539.469	−2.585	0.010
Cage	.038838	.012498	48,772.947	3.108	0.002
Cage^2^	−.015916	.004749	58,942.732	−3.351	0.001
40–44 years					
Constant term	9.125955	3.973687	52,292.011	2.297	0.022
[Sex = female]	1.718455	8.450069	63,836.054	0.203	0.839
Calendar year	0.000530	0.001978	52,290.339	0.268	0.789
Cage	−0.011926	0.005323	45,919.163	−2.240	0.025
Cage^2^	−0.000813	0.004207	63,833.556	−0.193	0.847
45–49 years					
Constant term	14.392008	3.666324	45,133.538	3.925	<0.0001
[Sex = female]	0.111541	0.028930	50,970.153	3.856	<0.0001
Calendar year	−0.002073	0.001825	45,147.299	−1.136	0.256
Cage	−0.011505	0.047202	36,973.118	−0.244	0.807
Cage^2^	−0.000515	0.003940	36,937.873	−0.131	0.896
50–54 years					
Constant term	21.134855	4.648196	28,090.963	4.547	<0.0001
[Sex = female]	0.081346	0.034127	35,679.933	2.384	0.017
Calendar year	−0.005912	0.002295	28,200.348	−2.576	0.010
Cage	0.142107	0.110024	22,499.549	1.292	0.197
Cage^2^	−0.006415	0.005025	22,452.308	−1.277	0.202
55–59 years					
Constant term	14.483520	6.509904	14,441.900	2.225	0.026
[Sex = female]	0.064112	0.042767	21,011.361	1.499	0.134
Calendar year	−0.003629	0.003124	14,741.530	−1.162	0.245
Cage	.381976	0.217497	10,973.387	1.756	0.079
Cage^2^	−0.013001	0.006831	10,958.017	−1.903	0.057
60–64 years					
Constant term	10.330497	10.184571	7038.354	1.014	0.310
[Sex = female]	0.066517	0.059201	10,801.639	1.124	0.261
Calendar year	−0.001449	0.004592	7487.328	−0.315	0.752
Cage	0.240891	0.427255	5330.091	0.564	0.573
Cage^2^	−0.006042	0.010224	5320.416	−0.591	0.555
65–69 years					
Constant term	−21.260625	17.377053	2734.553	−1.223	0.221
[Sex = female]	0.171801	0.083333	4650.236	2.062	0.390
Calendar year	0.010878	0.006919	3161.386	1.572	0.116
Cage	0.762101	0.807912	1984.811	0.943	0.346
Cage^2^	−0.015896	0.015617	1983.452	−1.018	0.309
70–74 years					
Constant term	−0.151714	38.355675	930.311	−0.004	0.997
[Sex = female]	0.132883	0.133375	1535.284	0.996	0.319
Calendar year	0.004731	0.012500	1203.265	0.379	0.705
Cage	0.066684	1.787135	737.826	0.037	0.970
Cage^2^	−0.002226	0.029030	738.639	−0.077	0.939
75–79 years					
Constant term	−88.263384	87.934236	243.989	−1.004	0.316
[Sex = female]	−0.080334	0.240411	417.653	−0.334	0.738
Calendar year	−0.000163	0.024665	404.215	−0.007	0.995
Cage	5.573218	4.115196	164.098	1.354	0.178
Cage^2^	−0.079425	0.057402	161.312	−1.384	0.168
80–94 years					
Constant term	269.841277	253.884634	29.281	1.063	0.297
[Sex = female]	−0.116419	0.532094	69.045	−0.219	0.827
Calendar year	−0.117305	0.057611	70.281	−2.036	0.046
Cage	−1.608711	10.503836	28.890	−0.153	0.879
Cage^2^	0.024360	0.128926	29.518	0.189	0.851
85–89 years					
Constant term	249.257018	1030.257290	15.000	0.242	0.812
[Sex = female]	−1.029095	0.987104	15.000	−1.043	0.314
Calendar year	0.103347	0.108753	15.000	0.950	0.357
Cage	−18.560493	42.777174	15.000	−0.434	0.671
Cage^2^	0.192158	0.470681	15.000	0.408	0.689

**Table 7:** Running speed (km/h, mean ± SD) for female age group marathoners

Year	18–24	25–29	30–34	35–39	40–44	45–49	50–54	55–59	60–64	65–69	70–74	75–79	80–84
1999	16.56 ± 3.56	15.48 ± 4.63	13.91 ± 3.96	13.77 ± 4.48	14.46 ± 3.91	13.29 ± 4.47	12.55 ± 3.25	12.16 ± 4.34	11.94 ± 2.32		10.65	7.90	
2000	14.11 ± 2.07	15.50 ± 4.53	15.02 ± 4.18	14.59 ± 4.27	14.42 ± 3.83	15.21 ± 3.98	12.68 ± 3.51	11.21 ± 3.99	10.02 ± 2.08	11.01 ± 1.34	10.82	10.57	
2001	12.32 ± 3.98	15.33 ± 4.06	14.36 ± 3.88	14.27 ± 4.23	14.02 ± 3.93	15.17 ± 4.19	12.82 ± 3.08	13.33 ± 3.13	11.59 ± 2.57	10.94 ± 2.15	11.76 ± 1.21		9.33
2002	14.21 ± 4.49	14.49 ± 3.73	14.40 ± 4.54	15.46 ± 4.14	14.63 ± 3.69	14.32 ± 4.13	12.83 ± 3.40	12.06 ± 3.50	12.94 ± 2.50	11.33 ± 1.83	10.98		
2003	16.54 ± 3.76	14.61 ± 4.19	15.02 ± 4.09	15.48 ± 4.10	15.08 ± 3.96	14.89 ± 4.15	12.70 ± 3.44	11.92 ± 3.38	11.19 ± 2.61	10.57 ± 2.10	10.54	10.36	
2004	15.43 ± 4.24	15.57 ± 4.04	14.68 ± 4.25	15.18 ± 4.07	15.42 ± 4.18	14.39 ± 3.88	12.71 ± 3.35	11.58 ± 3.48	11.37 ± 2.06	10.70 ± 1.30	10.29 ± 1.00	8.62	
2005	14.78 ± 4.72	15.52 ± 4.16	15.41 ± 4.05	14.26 ± 3.99	14.19 ± 4.02	14.68 ± 4.18	12.34 ± 3.22	11.54 ± 3.11	11.90 ± 2.83	10.00 ± 1.67	10.76 ± 2.18		
2006	15.64 ± 4.14	14.44 ± 4.06	15.02 ± 4.06	14.31 ± 4.03	14.62 ± 3.94	14.84 ± 4.22	12.86 ± 3.38	11.87 ± 3.35	11.27 ± 2.75	10.06 ± 1.69	9.21 ± 2.70	9.97	
2007	15.22 ± 4.52	15.47 ± 3.94	14.63 ± 4.09	14.79 ± 3.62	14.84 ± 4.14	13.03 ± 4.08	12.78 ± 3.04	11.21 ± 3.18	12.54 ± 2.66	11.06 ± 1.50	10.28 ± 2.80	9.54	
2008	14.79 ± 4.51	14.41 ± 4.07	14.73 ± 3.96	14.76 ± 4.15	14.01 ± 4.03	14.91 ± 4.26	12.79 ± 3.31	11.27 ± 3.31	11.98 ± 2.21	10.33 ± 1.97	10.28 ± 1.78	10.80 ± 1.94	9.13
2009	15.47 ± 3.89	15.02 ± 3.98	15.18 ± 4.08	14.74 ± 3.90	14.50 ± 3.95	14.52 ± 4.17	12.73 ± 3.27	11.79 ± 3.51	11.13 ± 2.02	11.50 ± 1.77	10.92 ± 2.64		
2010	14.44 ± 4.02	13.44 ± 4.33	14.13 ± 4.10	14.70 ± 4.17	14.51 ± 4.12	14.54 ± 4.28	13.69 ± 3.36	11.59 ± 3.54	11.49 ± 2.51	11.05 ± 1.55	11.41 ± 2.97	10.23 ± 1.72	
2011	15.36 ± 4.13	14.41 ± 4.13	15.02 ± 4.15	14.46 ± 4.04	14.61 ± 3.94	14.30 ± 4.09	15.37 ± 3.33	11.79 ± 2.26	11.93 ± 1.87	11.75 ± 2.63	10.32 ± 2.64	10.74	
2012	15.41 ± 4.39	14.06 ± 4.16	14.16 ± 4.09	14.14 ± 4.00	14.75 ± 4.06	14.33 ± 4.06	12.05 ± 3.11	11.19 ± 2.44	11.85 ± 2.55	11.51 ± 3.96	11.31 ± 0.98	10.04	
2013	15.88 ± 4.22	15.51 ± 4.07	14.88 ± 3.93	14.79 ± 3.97	14.99 ± 4.20	14.44 ± 4.12	12.28 ± 3.37	12.04 ± 2.45	11.27 ± 2.39	11.27 ± 4.29	10.59 ± 1.91	10.70	
2014	14.55 ± 4.41	15.28 ± 3.86	14.51 ± 3.98	14.17 ± 3.85	14.00 ± 4.12	14.01 ± 3.86	12.27 ± 3.19	11.50 ± 2.13	12.36 ± 2.31	12.03 ± 4.25	10.53 ± 2.36

**Table 8:** Running speed (km/h, mean ± SD) in male age group marathoners

Year	18–24	25–29	30–34	35–39	40–44	45–49	50–54	55–59	60–64	65–69	70–74	75–79	80–84	85–89	90–94
1999	16.34 ± 4.21	15.00 ± 3.92	13.86 ± 3.65	14.00 ± 4.03	13.86 ± 3.77	13.60 ± 3.78	13.24 ± 3.89	12.18 ± 3.25	11.25 ± 2.52	10.29 ± 3.19	10.04 ± 0.62	10.36			
2000	16.26 ± 3.94	14.66 ± 4.14	14.14 ± 4.21	14.47 ± 3.99	14.36 ± 3.80	14.05 ± 4.00	14.78 ± 4.15	12.88 ± 4.11	11.07 ± 2.81	10.83 ± 2.44	9.69 ± 2.98	10.48			
2001	14.35 ± 4.16	15.61 ± 4.06	14.01 ± 3.91	14.47 ± 3.83	14.01 ± 4.04	13.89 ± 4.08	14.33 ± 4.09	12.31 ± 4.00	11.02 ± 2.68	10.30 ± 2.61	10.73 ± 3.02	10.19	10.45		
2002	15.21 ± 4.28	14.89 ± 3.98	14.50 ± 3.91	14.70 ± 4.20	13.97 ± 3.86	14.38 ± 4.08	14.10 ± 4.11	12.82 ± 4.30	11.25 ± 2.17	10.43 ± 3.20	9.18 ± 2.91	10.83 ± 1.12			
2003	14.83 ± 4.20	15.19 ± 4.02	14.64 ± 3.97	14.25 ± 3.98	14.36 ± 3.98	14.44 ± 4.11	14.54 ± 4.31	12.92 ± 4.46	11.89 ± 2.81	10.98 ± 2.62	10.35 ± 2.25	11.70 ± 1.39	10.37		
2004	14.66 ± 4.23	14.93 ± 4.15	14.75 ± 3.94	14.24 ± 3.98	14.08 ± 4.05	13.91 ± 3.96	14.88 ± 4.11	12.72 ± 4.16	11.59 ± 2.16	11.02 ± 2.48	10.03 ± 2.91	10.30 ± 0.68			
2005	15.30 ± 3.97	14.82 ± 4.11	14.45 ± 4.00	14.59 ± 3.95	13.93 ± 3.85	14.26 ± 4.11	14.46 ± 4.20	12.23 ± 4.09	11.97 ± 2.65	10.50 ± 2.34	9.30 ± 2.79	10.09 ± 1.14			
2006	14.91 ± 4.39	14.87 ± 4.09	14.23 ± 3.92	14.49 ± 3.83	14.30 ± 3.97	14.49 ± 4.09	14.24 ± 4.15	12.65 ± 4.17	11.94 ± 2.47	10.65 ± 2.24	9.94 ± 1.38	9.24 ± 2.76	10.36		
2007	14.99 ± 4.20	14.80 ± 4.08	14.40 ± 3.96	14.61 ± 3.98	14.14 ± 3.95	14.30 ± 4.06	14.55 ± 4.27	12.59 ± 4.20	12.38 ± 2.25	11.23 ± 2.38	10.36 ± 2.21	10.16 ± 2.59	9.69 ± 0.98	10.54 ± 1.39	6.19
2008	15.23 ± 4.00	15.05 ± 4.03	14.49 ± 4.04	14.47 ± 4.10	14.00 ± 3.94	14.42 ± 4.06	14.65 ± 4.28	12.67 ± 4.34	12.06 ± 3.27	10.13 ± 2.24	10.88 ± 2.25	9.72 ± 2.52	9.39 ± 1.73		
2009	15.18 ± 4.23	14.82 ± 4.01	14.52 ± 3.96	14.55 ± 4.01	14.25 ± 4.02	14.34 ± 3.99	14.25 ± 4.25	12.91 ± 4.26	12.45 ± 2.25	10.78 ± 2.37	10.16 ± 2.23	10.13 ± 2.74	9.13 ± 1.18		
2010	15.33 ± 3.96	14.50 ± 4.13	14.31 ± 4.02	14.49 ± 4.09	14.45 ± 4.00	14.35 ± 4.14	14.24 ± 4.11	12.53 ± 4.23	12.23 ± 2.62	10.68 ± 2.53	9.95 ± 1.99	9.57 ± 2.96	10.87		
2011	15.33 ± 4.36	14.77 ± 4.10	14.63 ± 3.90	14.32 ± 4.10	14.30 ± 3.97	14.60 ± 4.09	14.33 ± 4.32	12.45 ± 4.21	12.95 ± 3.19	11.04 ± 2.47	10.42 ± 2.23	9.61 ± 2.10			
2012	14.75 ± 4.22	14.89 ± 4.06	14.25 ± 3.91	14.55 ± 3.96	13.93 ± 3.82	14.55 ± 4.05	14.25 ± 4.02	12.72 ± 4.43	12.29 ± 2.31	10.59 ± 3.23	9.79 ± 1.20	9.76 ± 2.97	9.76 ± 1.18		
2013	15.05 ± 4.35	15.42 ± 3.95	14.54 ± 4.00	14.43 ± 3.97	14.23 ± 4.09	14.42 ± 3.97	14.59 ± 4.06	12.99 ± 4.22	12.36 ± 3.11	11.14 ± 3.25	9.70 ± 2.30	10.07 ± 2.54		10.75 ± 1.71	
2014	14.91 ± 4.51	14.60 ± 4.10	14.58 ± 3.97	14.64 ± 3.89	13.97 ± 3.91	14.46 ± 4.05	13.84 ± 4.16	12.55 ± 4.23	12.03 ± 2.37	10.65 ± 2.41	9.89 ± 2.29	10.67 ± 1.13		10.12

**Table 9:** Results of the mixed-effects regression analyses for running speed in marathon

Parameter	Estimate	SE	df	t	*p*
18–24 years					
Constant term	54.963233	45.949509	1642.807	1.196	0.232
[Sex = female]	−0.010120	0.247367	1808.569	−0.041	0.967
Calendar year	−0.012919	0.022737	1694.390	−0.568	0.570
Cage	1.431063	0.966068	1517.761	1.481	0.139
Cage^2^	0.036707	0.024531	1541.276	1.496	0.135
25–29 years					
Constant term	35.772472	29.275418	3362.866	1.222	0.222
[Sex = female]	0.020400	0.169711	3644.906	0.120	0.904
Calendar year	−0.013980	0.014333	3395.444	−0.975	0.329
Cage	−1.058839	0.842978	2700.446	−1.256	0.209
Cage^2^	−0.037775	0.030204	2697.380	−1.251	0.211
30–34 years					
Constant term	18.697561	21.843554	4930.863	0.856	0.392
[Sex = female]	0.190907	0.136155	5546.306	1.402	0.161
Calendar year	−0.003024	0.010843	4923.040	−0.279	0.780
Cage	−0.481635	0.395727	3466.504	−1.217	0.224
Cage^2^	−0.027416	0.022019	3473.265	−1.245	0.213
35–39 years					
Constant term	24.985172	18.419663	5809.676	1.356	0.175
[Sex = female]	0.117198	0.120178	7035.058	0.975	0.329
Calendar year	−0.005163	0.009176	5811.848	−0.563	0.574
Cage	−0.070901	0.146207	4084.836	−0.485	0.628
Cage^2^	−0.010708	0.018256	4116.190	−0.587	0.558
40–44 years					
Constant term	24.560923	16.551235	6983.393	1.484	0.138
[Sex = female]	0.326742	0.110619	8241.252	2.954	0.003
Calendar year	−0.005067	0.008243	6983.203	−0.615	0.539
Cage	0.004824	0.037083	4870.786	0.130	0.896
Cage^2^	0.008351	0.016000	4750.139	0.522	0.602
45–49 years					
Constant term	−18.776608	18.688413	6055.809	−1.005	0.315
[Sex = female]	0.198713	0.121281	7183.064	1.638	0.101
Calendar year	0.016356	0.009307	6056.803	1.757	0.079
Cage	0.142027	0.219921	4077.826	0.646	0.518
Cage^2^	−0.008649	0.018296	4050.227	−0.473	0.636
50–54 years					
Constant term	35.122855	22.752641	3731.765	1.544	0.123
[Sex = female]	0.0376662	0.146151	5053.697	2.577	0.010
Calendar year	−0.013442	0.011249	3750.114	−1.195	0.232
Cage	1.138201	0.493626	2678.872	2.306	0.021
Cage^2^	−0.048973	0.022539	2671.667	−2.173	0.030
55–59 years					
Constant term	28.143217	31.538937	1687.963	0.892	0.372
[Sex = female]	0.426938	0.196830	2906.532	2.169	0.030
Calendar year	−0.007188	0.015146	1716.634	−0.475	0.635
Cage	0.096076	0.937454	1078.711	0.102	0.918
Cage^2^	−0.001525	0.029369	1070.383	−0.052	0.959
60–64 years					
Constant term	−42.833272	51.553601	1076.017	−0.831	0.406
[Sex = female]	0.496350	0.272019	1574.678	1.825	0.068
Calendar year	0.026103	0.023597	1184.052	1.106	0.269
Cage	0.567461	1.978831	738.994	0.287	0.774
Cage^2^	−0.014553	0.047523	749.129	−0.306	0.760
65–69 years					
Constant term	−59.473939	83.366094	418.223	−0.713	0.476
[Sex = female]	−0.231287	0.381408	737.633	−0.606	0.544
Calendar year	0.009479	0.034912	567.095	0.272	0.786
Cage	4.275632	3.531786	262.482	1.211	0.227
Cage^2^	−0.081406	0.068094	260.904	−1.195	0.233
70–74 years					
Constant term	−12.845200	143.465772	182.815	−0.090	0.929
[Sex = female]	−0.702660	0.576353	306.689	−1.219	0.224
Calendar year	−0.065044	0.051496	257.801	−1.263	0.208
Cage	10.243172	6.298387	109.487	1.626	0.107
Cage^2^	−0.163818	0.102047	109.268	−1.605	0.111
75–79 years					
Constant term	−60.658516	305.872037	10.992	−0.198	0.846
[Sex = female]	−0.568041	1.199866	84.162	−0.473	0.637
Calendar year	−0.087646	0.099109	43.052	−0.884	0.381
Cage	13.998502	14.954195	14.595	0.936	0.364
Cage^2^	−0.193455	0.209581	14.944	−0.923	0.371
80–84 years					
Constant term	−1994.108804	706.076437	15.000	−2.824	0.013
[Sex = female]	−1.193533	1.235529	15.000	−0.966	0.349
Calendar year	−0.064119	0.145369	15.000	−0.441	0.665
Cage	104.090501	24.280352	15.000	4.287	0.001
Cage^2^	−1.263960	0.295597	15.000	−4.276	0.001
85–89 years					
Constant term	−275.995974	516.465002	6.000	−0.534	0.612
[Sex = female]	0	0			
Calendar year	0.043759	0.245249	6.000	0.178	0.864
Cage	4.599196	1.543800	6.000	2.979	0.025
Cage^2^	0	0			

Sex difference in running speed decreased significantly in half-marathon running in age groups 35–39 and 50–54 years (Table 10) and in marathon running in age group 45–49 years (Table 11). For all other age groups, sex difference remained unchanged across years.

**Table 10:** Sex difference (%) in running speed in age group half-marathoners

Year	18–24	25–29	30–34	35–39	40–44	45–49	50–54	55–59	60–64	65–69	70–74	75–79	80–84	85–89
1999	1.4	−0.8	1.7	3.0	2.8	2.2	6.3	3.0	−1.3	−3.9	−11.1	−2.3		−49.0
2000	3.5	0.7	1.1	3.7	3.3	0.3	1.6	2.9	9.4	4.4	10.4	−7.2		
2001	−1.1	3.0	0.6	2.4	2.8	−0.7	0.4	0.8	2.0	−0.8	−11.1	12.8		
2002	−0.2	2.9	4.0	3.9	1.3	0.5	3.1	0.7	0.4	3.3	6.3	1.5	2.2	
2003	1.2	1.5	2.3	1.7	−1.6	3.5	2.5	1.5	5.3	6.7	7.4	13.7		
2004	1.7	3.4	1.3	2.6	0.8	0.3	2.7	1.3	−1.3	−2.8	15.0	−2.4	5.0	13.5
2005	−3.7	0.5	1.4	1.6	1.1	1.1	−0.6	−1.8	0.1	1.2	−2.3	21.8	1.5	
2006	−0.9	−2.2	0.1	2.5	−0.06	0.9	0.7	0.09	−1.8	−3.1	11.7	−25.2		
2007	−0.8	−0.2	−0.6	1.1	−3.2	−0.8	−0.6	0.2	−1.4	−1.7	−11.8	−0.6	−7.9	−22.7
2008	−0.2	−0.8	1.6	1.6	−0.6	1.4	−0.7	−0.7	−1.0	2.6	3.1	−0.2	−34.2	
2009	−0.4	−0.1	1.9	0.03	0.6	1.5	1.1	2.1	0.9	3.3	3.8	28.9	−22.4	
2010	0.5	0.7	2.4	0.03	1.2	−0.5	1.7	0.7	1.2	4.2	3.4	−10.4	−9.3	
2011	−0.8	0.9	1.5	1.6	1.5	1.3	−1.4	1.2	0.1	5.8	−6.2	−7.3	9.2	
2012	2.4	−1.1	2.3	0.4	0.8	0.5	0.5	2.8	0.4	3.6	0.7	2.1	−11.6	7.9
2013	1.5	1.5	−0.3	−0.4	0.4	−0.1	0.4	0.4	1.7	−0.6	4.1	−3.1	−3.4	
2014	0.5	−0.0	0.1	0.1	−0.02	−0.1	−0.5	−0.2	0.01	1.1	0.07	−9.0		
r^2^	0.001	0.08	0.06	0.73	0.145	0.06	0.39	0.07	0.08	0.04	0.0001	0.02	0.05	0.44
*p*	0.89	0.26	0.34	<0.0001	0.14	0.33	0.0092	0.31	0.27	0.42	0.97	0.55	0.51	0.33

**Table 11:** Sex difference (%) in running speed in age group marathoners

Year	18–24	25–29	30–34	35–39	40–44	45–49	50–54	55–59	60–64	65–69	70–74	75–79	80–84
1999	1.3	3.1	0.4	−1.6	4.3	−2.2	9.9	−13.3	10.4		−7.7	−59.1	
2000	−37.8	5.7	6.2	0.8	0.4	8.2	−0.6	2.2	−14.5	−40.4	28.0	19.9	
2001	−14.1	−1.8	2.5	−1.3	0.1	9.1	3.4	14.0	−9.5	−14.4	−16.7		−0.6
2002	−6.5	−2.6	−0.6	5.1	4.7	−0.3	5.1	−5.1	−17.4	−0.6	−13.6		
2003	11.4	−3.8	2.5	8.6	5.0	3.1	1.1	−6.6	8.7	10.5	−17.1	56.9	
2004	5.2	4.3	−0.4	6.6	9.4	3.4	−1.1	5.8	12.1	−2.1	30.3	−55.3	
2005	−3.4	4.6	6.6	−2.2	1.9	3.0	−0.8	9.2	6.1	10.3	−27.8		
2006	4.9	−2.9	5.5	−1.2	2.2	2.4	4.3	8.2	2.2	2.6	−24.9	15.8	
2007	1.5	4.5	1.6	1.2	4.9	5.1	1.6	4.2	−5.8	−1.1	5.6	−44.4	
2008	−2.9	−4.2	1.6	1.9	0.1	3.3	0.9	4.0	−0.5	1.2	15.1	−16.4	−18.7
2009	1.8	1.3	4.5	1.3	1.8	1.2	3.3	5.9	4.4	−1.9	−1.5		
2010	−5.8	−7.3	−1.2	1.4	0.4	1.3	10.1	7.2	8.3	2.3	−32.6	45.1	
2011	0.2	−2.4	2.6	0.9	2.1	−2.0	7.2	2.3	−0.1	−1.8	13.1	21.4	
2012	4.4	−5.6	−0.6	−2.7	5.9	−1.4	5.6	−3.6	10.1	−13.3	−7.8	13.6	
2013	5.5	0.5	2.3	2.4	5.3	0.1	4.7	0.3	−0.6	−11.5	18.4	43.0	
2014	−2.4	4.6	−0.5	−3.2	0.1	−3.1	3.0	6.5	8.8	2.6	−9.1		
r^2^	0.15	0.05	0.05	0.05	0.006	0.28	0.04	0.05	0.12	0.06	0.0009	0.16	1
*p*	0.13	0.37	0.36	0.36	0.76	0.033	0.45	0.39	0.18	0.37	0.91	0.21	

### Age

**Fig. 3:**
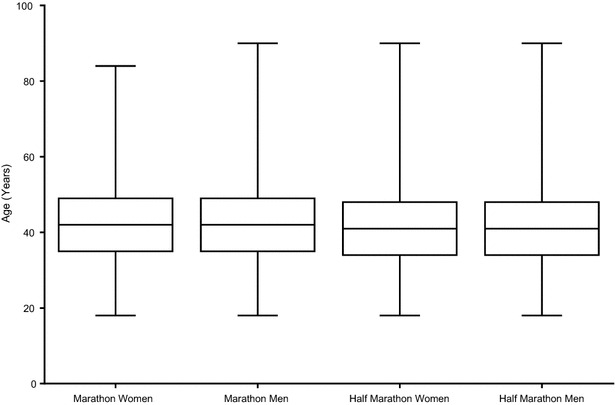
Box–whisker-plot for age in female and male half-marathoners and marathoners. Data are presented as mean ± 95 % confidence interval

Figure 3 shows the box–whisker-plots for age for female and male half-marathoners and marathoners. In marathoners, women (42.18 ± 10.63 years) were at the same age than men (42.06 ± 10.45 years) (p > 0.05). Similarly, in half-marathoners, women (41.40 ± 10.63 years) were at the same age than men (41.31 ± 10.30 years) (p > 0.05). However, women in marathon running were 0.78 ± 0.33 years older than women in half-marathon running (p < 0.001) and men in marathon running were 0.75 ± 0.14 years older than women in half-marathon running (p < 0.001).

**Fig. 4:**
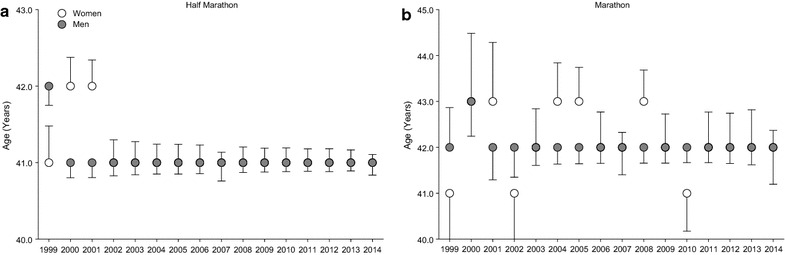
Age across years in half-marathoners and marathoners. Data are presented as mean ± SD. From 2002 to 2014, data for women are hidden behind data for men

Figure 4 shows trend in age of half-marathoners and marathoners across years. In female (r^2^ = 0.00, p = 0.93) and male (r^2^ = 0.12, p = 0.18) marathoners, age remained unchanged. Similarly, in female (r^2^ = 0.12, p = 0.19) and male (r^2^ = 0.06, p = 0.34) half-marathoners, age remained unchanged across years.

## Discussion

This study intended to compare participation, performance and age of half-marathoners and marathoners competing in Switzerland between 1999 and 2014. The most important findings were: (1) more athletes competed in half-marathons than in marathons, (2) women were running faster than men in both half-marathons and marathons, (3) half-marathoners were running slower than marathoners, and (4) half-marathoners were younger than marathoners.

### Higher participation in half-marathons compared to marathons

A first important finding was that 12.3 times more women and 7.5 times more men competed in half-marathon running than in marathon running, respectively; that was, an overall 8.7 half-marathon to marathon runner’s ratio. This ratio was quite higher than the ratio of 3.71 which can be calculated from the data of the USA (www.runningusa.org/2015-national-runner-survey) for the year 2014.

Thus, this ratio might vary from country to country and by gender, as a higher ratio was observed in women. For example, in the USA, the percentages of female and male half-marathoners in 2014 were 61 and 39 %, respectively (www.runningusa.org/half-marathon-report-2015). For marathoners in the same year and the same country, the percentages were, however, 43 and 57 %, respectively (www.runningusa.org/marathon-report-2015). In other terms, 1.56 times more women competed in half-marathon running, but 1.32 times more men in marathon running in 2014 in the USA. When we compare the 2014 data of the USA to the data from 1999–2014 in Switzerland, 10.2 times more marathoners competed in the USA (550,637) compared to Switzerland (53,694). Considering the sexes, there were 23.2 times more women and 7.2 times more men in the USA than in Switzerland. For half-marathon running, there were 4.5 times more athletes in the USA (2046,600) compared to Switzerland (454,324). There were 9.9 times more women and 2.4 times more men in the USA compared to Switzerland. Considering the data of Leyk et al. (2007) investigating 65 half-marathons and 69 marathons held in Germany between 2003 and 2005, a total of 156,717 men and 144,640 women were considered with a ratio of 1.08. There were 4.85 and 2.6 times more men in marathons and half-marathons, respectively (Leyk et al. 2007). When we compare their data to the data from Switzerland, we had 2.9 times fewer marathoners, but 3.1 times more half-marathoners. In details, we considered 2.6 times fewer male (43,489 vs. 129,929) and 2.98 fewer female (10,205 vs. 26,788) marathoners. For half-marathoners, we investigated 3.1 times more female half-marathoners (125,894 vs. 39,998) and 3.1 times more male half-marathoners (328,430 vs. 104,042). Obviously, the data set and the country seems to have an influence on the participation trends in half-marathon and marathon running.

An interesting observation was that participation increased across years in both half-marathon and marathon running. When the Swiss data were investigated from 2000 to 2010, the number of half-marathoners increased significantly for both men and women. In contrast, the number of male and female full marathoners increased until 2005 only and decreased thereafter (Anthony et al. 2014). Most probably, after 2010, a new increase (hype) in marathon running occurred in Switzerland, which might also explain the better performance in marathon running compared to half-marathon running.

### Women were faster than men

A second important and unexpected finding was that women were significantly faster than men in both half-marathon and marathon running. However, the differences were very small, but still significant. This finding was not in agreement with a previous study, in which female long-distance runners were slower by 22.5 and 20 % in half-marathon and marathon running, respectively, than men (Leyk et al. 2007). A potential explanation could the sample size. While Leyk et al. (2007) considered 405,515 race times, we analyzed 508,108 race times (i.e. 25.3 % more athletes).

An explanation of a superior performance in men might be their training characteristics, as a research on the these characteristics of the 2004 USA Olympic marathon trials qualifiers showed that men and women ran 75 and 68 % of their weekly training distance, respectively, below marathon race pace, and men had more years of sports experience, ran more often and ran farther (Karp 2007). It has been shown that in men, the mean weekly running distance, the minimum distance run per week, the maximum distance run per week, the mean weekly hours of running, the number of running training sessions per week, and the mean speed of the training sessions were significantly and negatively related to total race time, but not in women (Knechtle et al. 2010).

A potential explanation for the disparate findings in Leyk et al. (2007) and our findings for female and male running performance could be the kind of analysis. While Leyk et al. (2007) compared in their study the top ten half-marathon and marathon race times in women and men, women were on average 20 % slower in marathon and 22.5 % slower in half-marathon. In the present study, however, women were faster than men when all recorded women and men were considered for data analysis. While Leyk et al. (2007) investigated 104,042 male and 39,998 female half-marathoners and 129,929 male and 26,788 female marathoners, our numbers were 328,430 male and 125,894 female half-marathoners and 43,489 male and 10,205 female marathoners. In fact, we considered 3.15 times more half-marathoners (i.e. 3.15 times more men and 3.14 times more women) but 2.9 times fewer marathoners (i.e. 2.98 fewer men and 2.62 times fewer women).

The considerably higher number of half-marathoners might explain why they were significantly slower than marathoners. In a large number of athletes, also slow to very slow runners are included. Similarly, the lower number of marathoners might be a selection of faster runners. Most probably, more recreational runners compete in Switzerland in half-marathons and more elite runners in marathons. This assumption might be supported by the data from 2014 in the USA where 3.71 more female and male runners competed in half-marathons (www.runningusa.org/half-marathon-report-2015) compared to marathons (www.runningusa.org/marathon-report-2015). There were more women (61 %, 1,248,426) than men (39 %, 798,174) competing in half-marathons but more men (57 %, 313,863) than women (43 %, 236,774) in marathons. In half-marathon races, women (2:21 h:min) were running 0:19 h:min (7.42 %) slower than men (2:02). In marathon races, women (4:19 h:min) were running 0:25 h:min (11.36 %) slower than men (4:19 h:min). A further explanation for the different findings between Leyk et al. (2007) and our findings could be the period of time. While Leyk et al. (2007) considered marathons in Germany held from 2003 to 2005 (i.e. 3 years) we included marathons in Switzerland held from 1999 to 2014 (i.e. 15 years). Across years, women were able to improve their running performance.

Nevertheless, the present study was not the first one to observe a superior performance in women during an endurance event. Recently, a superior performance of women was noticed in ultra-distance swimming (Knechtle et al. 2014, 2015a), which might be attributed to anthropometric characteristics such as body fat.

### Half-marathoners are running slower than marathoners

A third important finding was that female and male half-marathoners were running slower than female and male marathoners. This might be explained by their pre-race preparation, their sport experience and their competitive level. In a field study comparing 147 recreational male half-marathoners and 126 recreational male marathoners, the half-marathoners were running for fewer years, completed less weekly running kilometers, they were running fewer hours per week, completed fewer training sessions, achieved fewer kilometers per training session, and invested fewer minutes per training session compared to the marathoners (Zillmann et al. 2013). However, in that study, the half-marathoners (12.2 ± 1.9 km/h) were running significantly faster than the marathoners (11.1 ± 1.4 km/h). This might be explained by the fact that the subjects could participate in that study voluntarily and the interests to take part in such an investigation might be different for half-marathoners and marathoners.

Another potential bias could be the race fee and/or average yearly income in USA, Switzerland and Germany. The race fee in a half-marathon is lower than in a full marathon. For example, the entry fee for running the half-marathon in ‘Lausanne Marathon’ is 52 Swiss Francs, but 80 Swiss Francs for running the full marathon (http://de.lausanne-marathon.com/inscription/inscriptions/prix-categories/). While the annual income is higher in the USA compared to Germany, the income in Switzerland is higher compared to Germany. In Switzerland, the average household net-adjusted disposable income per capita is USD 33, 491 a year (www.oecdbetterlifeindex.org/countries/switzerland). In comparison, the average household net-adjusted disposable income per capita is USD 31,252 in Germany (www.oecdbetterlifeindex.org/countries/germany). In the USA, however, the income is higher with USD 41,355 (www.oecdbetterlifeindex.org/countries/united-states).

Differences in performance between half-marathoners and marathoners might be due to differences in anthropometric characteristics. For instance, with regards to anthropometric characteristics, male half-marathoners were heavier with longer legs, thicker upper arms and thigh, and higher skinfold thicknesses, body fat percentage and skeletal muscle mass compared to male marathoners (Zillmann et al. 2013). Compared to ultra-marathoners, female half-marathoners were younger, heavier, reported a lower training volume and had a lower incidence of bone stress injury (Micklesfield et al. 2007). Race time in half-marathon running might be predicted by body mass index, resting heart rate, training volume and sport experience (Campbell, 1985). In addition to anthropometric and training characteristics, the performance in half-marathon running has been shown to be influenced by certain physiological parameters. In a small group of female and male half-marathoners, their race speed corresponded to ~79 % of VO_2_max and their race time correlated to VO_2_max and running speed at blood lactate concentration 4 mmol · l^−1^ (Williams and Nute, 1983). In a comparison of female middle- and long-distance runners, the race time in half-marathon running correlated to body mass, but not to VO_2_max, anaerobic threshold or running economy (Nurmekivi et al. 1998). Performance in marathon running has been shown to be limited by the rate of aerobic metabolism of a limited amount of carbohydrate energy and the velocity that can be maintained without developing hyperthermia (Coyle, 2007). Serum leptin, which decreases in the blood when the energy balance is negative, lowered after an ultra-marathon race, but not after a half-marathon (Zaccaria et al. 2002). In a genetic study of ACE I/D polymorphism, no association between half-marathoners and the ACE genotype was found, whereas an increase of the I/I genotype incidence in the successful marathoners was observed (Hruskovicová et al. 2006).

### Half-marathoners were younger than marathoners

A fourth important finding was that female and male half-marathoners were younger than female and male marathoners. Within a race distance, no differences were found between the sexes. These findings were different to the study subjects in the field study of Zillmann et al. (2013). There, the age of male half-marathoners was 40.2 ± 10.1 and 42.8 ± 10.8 years for male marathoners. However, the difference of ~2.6 years was not statistically significant.

Similarly, in US-American half-marathoners (www.runningusa.org/half-marathon-report-2015) and marathoners (www.runningusa.org/marathon-report-2015), the ages were different. In marathoners competing in the USA in 2014, women (~40.0 years) were on average ~4.0 years older than men (~36.0 years). In half-marathoners, women (~36.0 years) were ~3.1 years younger than men (~39.1 years). Therefore, female half-marathoners were ~4.0 years younger than female marathoners, and male half-marathoners were ~3.1 years older than male marathoners. The differences might be explained that in the study of Zillmann et al. (2013) only athletes competing in one marathon in Switzerland were examined and in the statistic report in the USA, the median age of all successful finishers was provided (www.runningusa.org/statistics).

A further observation was that women were at about the same age than men in marathon (~42 years) and half-marathon (~41 years) running. However, women in marathon running were significantly older than women in half-marathon running and men in marathon running were significantly older than women in half-marathon running. Generally, elite marathoners are considerably younger than the athletes in the present sample. In the study of Hunter et al. (2011) investigating the first five placed women and men competing in marathons of the ‘World Marathon Majors Series’ in Berlin, Boston, Chicago, London, New York City, the International Athletic Association Federation (IAAF) World Championships, and the Olympic Games, women (29.8 ± 4.2 years) were older than men (28.9 ± 3.8 years), but for only two (i.e. Chicago and London) of the seven marathons with no sex difference in age for the marathons held in Berlin, Boston, New York City, and at the IAAF World Championships and the Olympic Games.

The age of the best marathon performance is, however, higher in recreational runners. In a study investigating male amateur runners competing in the Stockholm Marathon between 1979 and 2014, marathon race performance of the average runner improved up to age of 34.3 ± 2.6 years. After that age, the marathon race performance started to decline (Lehto 2015). The differences between the age might be explained in that Hunter et al. (2011) investigated elite marathoners competing at world class level while we considered all successful finishers in half-marathon and marathon running.

### Limitations

A limitation of the present study was the lack of information with regards to the competitive level of runners, i.e. whether they were elite or recreational athletes. The possibility that women were more competitive than men and overall marathoners more competitive than overall half-marathoners cannot be excluded and might account for the better performance in women and in marathoners, respectively. On the other hand, data on more than half a million runners were examined in this study which was one of the largest samples of half-marathoners and marathoners ever studied.

## Conclusions

In summary, for runners competing between 1999 and 2014 in Swiss half-marathons and marathons, (1) more athletes competed in half-marathons than in marathons, (2) women were running faster than men, (3) half-marathoners were running slower than marathoners, and (4) half-marathoners were younger than marathoners.
